# Multivitamin consumption and childhood asthma: a cross-sectional study of the NHANES database

**DOI:** 10.1186/s12887-024-04540-5

**Published:** 2024-01-31

**Authors:** Li Zhang, Yali Xu, Xuemei Li, Fan Yang, Chengxiu Wang, Chunmei Yu

**Affiliations:** 1 Department of Pediatrics, Women and Children’s Hospital of Chongqing Medical University, Chongqing, 401147 P.R. China; 2 Department of Pediatric Center, The Third Affiliated Hospital of Chongqing Medical University, Chongqing, 401120 P.R. China; 3 Department of Pediatrics, The Fifth People’s Hospital of Chongqing, No.24 Renji Road, Nanan District, Chongqing, 400062 P.R. China

**Keywords:** Dietary, Multivitamin, Childhood asthma, NHANES, Bayesian kernel machine regression

## Abstract

**Background:**

Dietary intakes of vitamins are associated with asthma. However, previous studies mainly explored the association between a single vitamin intake and asthma, which did not take the multivitamins into consideration. Herein, this study aims to explore the overall effect of dietary multivitamins consumption on childhood asthma.

**Methods:**

Data of children and adolescents (aged 2-17 years old) were extracted from the National Health and Nutrition Examination Survey (NHANES) database in 2015-2018 in this cross-sectional study. Weighted univariate logistic regression analysis was used to screen covariates. The association between multivitamins (including vitamin A, C, D, E, B1, B2, B6, B12, K, niacin, folic acid, and choline) and childhood asthma was explored using univariate and multivariate logistic regression analyses. The evaluation indexes were odds ratio (OR) and 95% confidence interval (CI). We further introduced the Bayesian kernel machine regression (BKMR) to assess the joint effect of the twelve vitamins on childhood asthma, the impact of an individual vitamin as part of a vitamin mixture, and the potential interactions among different vitamins.

**Results:**

Among 4,715 eligible children and adolescents, 487 (10.3%) had asthma. After adjusting for covariates including race, family history of asthma, pregnant smoking, BMI Z-score, energy intake, breast feeding, and low birth weight, we found that for each 1-unit increase in vitamin K consumption, the odds of childhood asthma decreased 0.99 (*P*=0.028). The overall effect analysis reported a trend of negative relationship between the multivitamins and childhood asthma, especially at the 75th percentile and over. According to the BKMR models, when other vitamins are fixed at the median level, the odds of childhood asthma increased along with the elevated vitamin D (VD) and vitamin B2 (VB2), whereas along with the depressed vitamin C (VC). In addition, no potential interaction has been found between every two vitamins of multivitamins on childhood asthma.

**Conclusion:**

Among children and adolescents who have high-risk of asthma, it may be beneficial to increase dietary consumption of multivitamins. Our findings recommended that children and adolescents should increase the intake of VC-rich foods, whereas control the dietary consumption of VD and VB2 in daily life.

## Background

Asthma is one of the most common chronic diseases of childhood, and the disease burden is in a rising trend globally [1, 2]. The mean worldwide prevalence of asthma in 2020 was about 13% in adolescents and 11% in children [3]. Although inhaled corticosteroids are the most effective and commonly used treatment for persistent asthma [4], some of the children may suffer from frequent and severe asthma attacks, causing deterioration of lung function [5], and thus increasing the psychological burden on both children and parents [6].

Diet is a common modifiable factor related to the airway inflammation and asthma [7]. High dietary inflammatory index has been reported to be associated with high-burden of childhood asthma [8]. Vitamins are obtained from the daily diet and play important roles in maintaining normal physiological functions of the human body, and take part in the occurrence and development of a variety of diseases [9]. Anti-oxidative vitamins, such as vitamin C (VC) and vitamin E (VE), have anti-inflammation effects and are involved in the oxidative stress caused by scavenging reactive oxygen species (ROS), and thus may reduce the risk of asthma [10, 11]. On the other hand, dietary intake of a number of vitamins may be associated with an increased risk of asthma. A Lasso regression analysis conducted by Qu et al. [12] found that dietary vitamin B12 (VB12) consumption was positively associated with childhood asthma. Vitamins including folic acid, VB12, and choline are the common methyl donors of human DNA methylation, which may promote the risk of allergic airway diseases [13, 14]. However, existing researches have mainly explored the association between a single vitamin intake and childhood asthma, which not take multivitamins into consideration.

Actually, human beings are exposed to multiple dietary vitamins, and there may be potential synergistic or antagonistic effects between different vitamins on diseases’ progression. Herein, the current study aims to explore the overall effect of dietary consumption of multivitamins on childhood asthma, and hope our findings could provide some information on diet management in children and adolescents who are at high-risk of asthma.

## Methods

### Study design and population

Data of children and adolescents in this cross-sectional study were extracted from the National Health and Nutrition Examination Survey (NHANES) database in 2015-2018. The NHANES database is designed to sample the non-institutionalized population in the United States using a complex, multistage stratified probability sample based on selected counties, segments, households, and individuals. For more details, please visit the NHANES website: https://www.cdc.gov/nchs/nhanes/index.htm. The National Center for Health Statistics (NCHS) well trained professionals conducted interviews in participants’ homes, and extensive physical examinations were conducted at mobile exam centers (MECs).

A total of 9,645 children and adolescents (aged 2-17 years old) were initially included. The exclusion criteria were (1) missing information on asthma diagnosis, (2) missing the information on dietary intake (collected by the first 24-hour recall), and (3) missing information of body mass index (BMI) Z-score. Finally, 4,715 were eligible. The NHANES was approved by the Institutional Review Board (IRB) of the United States NCHS and Centers for Disease Control and Prevention (CDC). All study methods were performed in accordance with the relevant guidelines and regulations. Since the NHANES database is publicly available, and written informed consent has been obtained from participants before any data collection, no ethical approval of the IRB of The Fifth People’s Hospital of Chongqing was needed.

### Measurement of multivitamin consumption

Dietary intake of vitamins and their supplements were collected using two 24-hour dietary recalls surveys in the NHANES [15]. The first 24-hour recall interview was conducted in person in the MEC by trained interviewers, and the second interview was performed by telephone or mail 3-10 days later. The food groups were categorized using the United States Department of Agriculture (USDA) Food and Nutrient Database for Dietary Studies 2.0. Duration and quantity of multivitamins were assessed among participants who reported taking any prescription or non-prescription supplements in the past 30 days. In this study, we used the information of the first 24-hour dietary recall on vitamins including vitamin A (VA), VC, vitamin D (VD), VE, vitamin B1 (VB1), vitamin B2 (VB2), niacin, vitamin B6 (VB6), VB12, folic acid, choline.

### Assessment of childhood asthma

The childhood asthma was diagnosed using self-reported questionnaires, which was recorded in the “Medical Conditions” section in the NHANES interview. Asthma was defined by respondents giving positive responses to both following two questions (by proxy if under age 16): “Has a doctor or other health professional ever told you that you have asthma?” and “In the past 12 months, have you had wheezing or whistling in your chest?”. Only children who still had asthma at the time of during the process of this study were included in the asthma group.

### Variables collection

We collected variables including age (divided into 2-6, 7-11, and 12-17 years old) [16], gender, race, poverty-to-income ratio (PIR) (divided into <1, and ≥1) [17], household smoke exposure, family history of asthma, physical activity, sedentary time, pregnant smoking, breast feeding, low birth weight, BMI Z-score, and energy intake from the NHANES database.

Low birth weight was defined as weight <2500 g [18]. The weight status of children and adolescents aged 2-17 years old was assessed by the BMI Z-score. The BMI z-score is the CDC recommended percentiles, which was calculated accounting for age and sex. For more details of the calculation on BMI z-score please visit the NHANES website: https://www.cdc.gov/growthcharts/html_charts/bmiagerev.htm. Household smoke exposure (i.e., family members’ smoking status) was estimated by the NHANES family questionnaire (SMQFAM: SMD460). Serum cotinine concentration was used to reflect the tobacco smoke exposure. A participant with a serum cotinine level above 10 ng/mg was recognized as a smoker. If a participant he or she self-identified as living in a home in which someone smoked and had a cotinine level greater than 0.011 ng/mg but less than or equal to 10 ng/mg was defined as having second hand smoke exposure. A participant was defined as having tobacco smoke exposure of unknown source if he or she indicated he or she did not live in a home in which someone smoked and had a cotinine level greater than 0.011 ng/mg but less than or equal to 10 ng/mg. Individuals with non-detectable serum cotinine levels were defined as non-smokers [19, 20]. Having a family history of asthma was defined as those who responded “yes” to the question: “Among family members living and deceased, were any of (subject's/your) close biological relatives (i.e., blood relatives) such as father, mother, sisters or brothers, ever told by a health professional that they had asthma”?

The Global Physical Activity Questionnaire (PAQ) was used to assess the physical activity levels of NHANES participants [21]. This questionnaire consisted of the frequency (the times of days per month/per week) and the duration spent on the item during 30 days or 7 days. Metabolic equivalent (MET) is the unit that measures the energy expenditure after the participants have completed a specific activity. Energy expenditure (MET·min) = recommended MET × exercise time of corresponding activity (min). Each physical activity of children and adolescents was estimated by multiplying the number of days by the average time by the corresponding MET and then summing up the obtained values to calculate the total MET for each participant. The ideal physical activity was defined as ≥180 MET·min/day or ≥60 min/day in children who aged 2-11 years old [22]. Sedentary time (time watching TV or video or using a computer) per average day over the last 30 days was also asked in the household interview. The classification for sedentary time was based on total time spent watching TV or videos and using computers per day for children and adolescents (<3, 3-6, and ≥6 hours).

### Statistical analysis

Normal distributed data was expressed as mean and standard error (Mean ± S.E), and t test was used for comparison between two groups. Categorical data were described by the frequency and constituent ratio [N (%)], and chi-square test (χ2) was used for the comparison. The MEC 2-year cycle weight (WTMEC2YR) was used for statistical analyses. The NHANES provides a set of special weights (WTDRD1) for an analysis that uses the data of the first 24-hour dietary recall. The WTDRD1 weights were constructed by taking the MEC sample weights (WTMEC2YR) and further adjusting for (a) the additional non-response and (b) the differential allocation by day of the week for the dietary intake data collection. More details could been found elsewhere: https://wwwn.cdc.gov/Nchs/Nhanes/2005-2006/DR1IFF_D.htm#WTDRD1.

Weighted univariate logistic regression analysis was used to screen the covariates, and those significantly associated with childhood asthma (*P* value <0.05) were then included in the adjustment of multivariate models. The association between multivitamins consumption and childhood asthma were explored using weighted univariate and multivariate logistic regression analyses. The multivariate models adjusted for age, race, family history of asthma, pregnant smoking, BMI Z-score, breast feeding, low birth weight, and energy intake.

Considering the possible nonlinearity and non-additive effects among mixture vitamins, we introduced the Bayesian kernel machine regression (BKMR) to assess (1) the joint effect of twelve vitamins on the childhood asthma, (2) the impact of each individual vitamin as part of the vitamin mixture, and (3) the potential interaction among different vitamins [23, 24]. We used a probit link function with BKMR in consideration of present binary outcome (asthma or non-asthma) [23]. The Pearson correlation coefficients were used to classify these vitamins into different groups. Considering the relatively high correlations among vitamins, we further implemented a hierarchical variable selection method with 50,000 iterations using a Markov chain Monte Carlo algorithm. We then calculated the group posterior inclusion probability (groupPIP) and the conditional posterior inclusion probability (condPIP).

The evaluation indexes were the odds ratios (ORs) and 95% confidence intervals (CIs). Two-sided *P*<0.05 was considered significantly associated. R version 4.2.2 (Institute for Statistics and Mathematics, Vienna, Austria) and Python 3.9.0 (Python Software Foundation, Delaware, USA) were used for analysis. The variables included missing values, including education level and cotinine, were deleted because their deficiency proportion were more than 20%.

## Results

### Characteristics of study participants

**Fig. 1:**
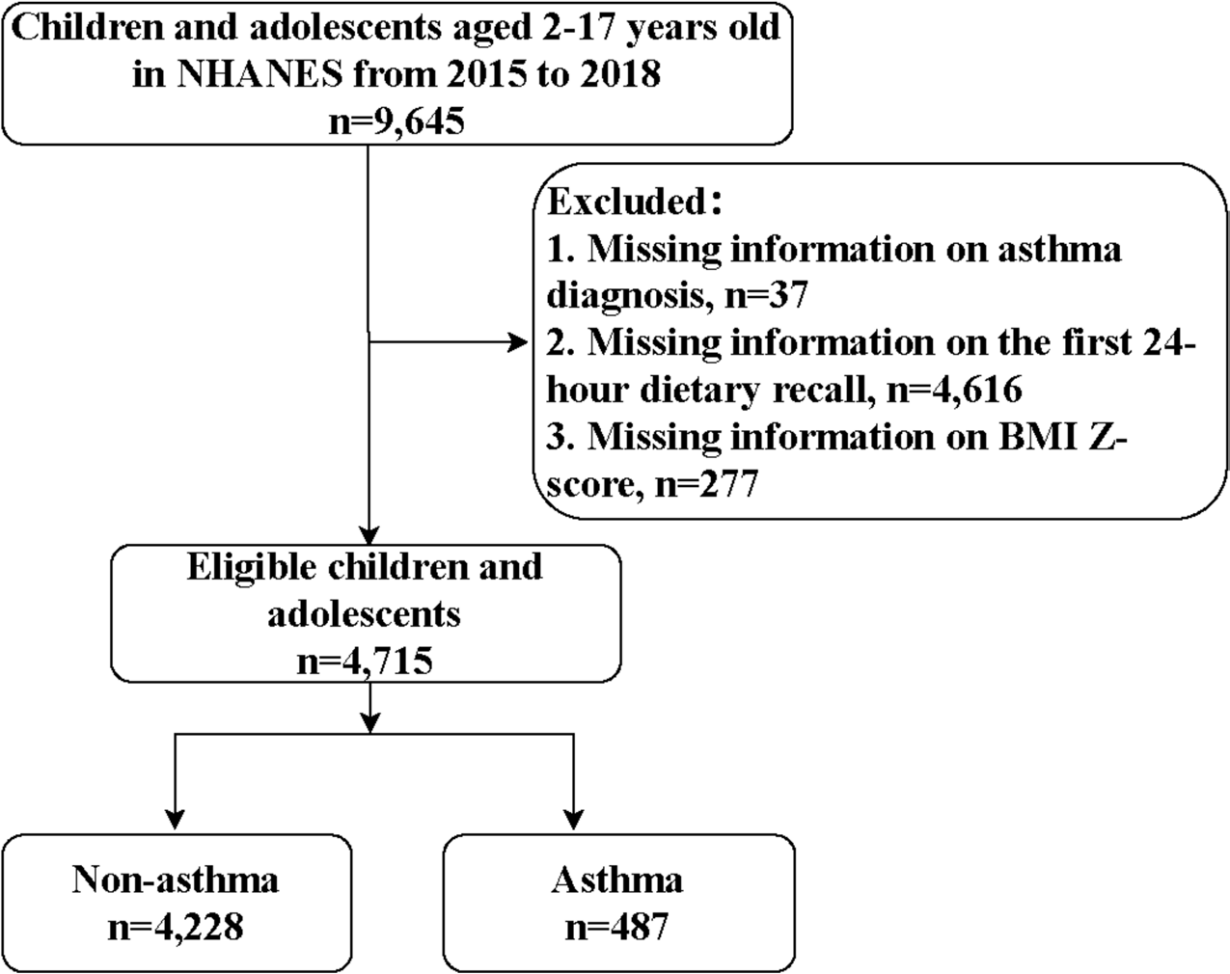
Flowchart of the participants screening

Figure 1 was the flowchart of participants screening. We initially included 9,645 children and adolescents who aged 2-17 years old in the NHANES from 2015 to 2018. We excluded participants without the information on asthma diagnosis (*n*=37), first 24-hour dietary recall (*n*=4616), or BMI Z-score (*n*=277). Finally, 4,715 were eligible.

Table 1 compares the characteristics of eligible children and adolescents between asthma group and non-asthma group. A total of 487 (10.3%) children and adolescents had asthma. The average age of study population was 10.1 years old, and 1,482 (53.2%) were White. There were 1,195 (27.0%) children and adolescents had family history of asthma. The numbers of who had breast feeding and low birth weight were respectively 1,091 (21.2%) and 572 (11.1%). The average dietary VC consumptions were respectively 77.53 mg and 68.65 mg in asthma group and non-asthma group. The mean BMI Z-score between asthma group and non-asthma group were respectively 0.90 and 0.59. In addition, energy intake was significantly different between asthma group (2037.80 kcal) and non-asthma group (1890.63 kcal).

**Table 1:** Comparation between asthma group and non-asthma group in characteristics of children and adolescents

Variables	Total (*n*=4715)	Asthma (*n*=487)	Non-asthma (*n*=4228)	*P*
Age, year, Mean (S.E)	10.10 (0.10)	10.59 (0.25)	10.04 (0.11)	0.054
Age, n (%)				0.017
2-6	1434 (26.9)	104 (19.6)	1330 (27.7)	
7-11	1544 (29.4)	193 (33.7)	1351 (28.9)	
12-17	1737 (43.7)	190 (46.7)	1547 (43.4)	
Gender, n (%)				0.107
Male	2348 (50.5)	275 (55.3)	2073 (50.0)	
Female	2367 (49.5)	212 (44.7)	2155 (50.0)	
Race, n (%)				<0.001
White	1482 (53.2)	138 (48.2)	1344 (53.8)	
Black	1065 (13.0)	161 (20.9)	904 (12.1)	
Other	2168 (33.8)	188 (30.9)	1980 (34.1)	
PIR, n (%)				0.223
< 1	1226 (19.6)	144 (23.3)	1082 (19.2)	
≥ 1	3101 (73.9)	302 (70.0)	2799 (74.3)	
Unknown	388 (6.6)	41 (6.7)	347 (6.6)	
Household smoke exposure, n (%)				0.399
Yes	1352 (27.1)	149 (29.1)	1203 (26.9)	
No	109 (1.8)	13 (2.6)	96 (1.7)	
Unknown	3254 (71.1)	325 (68.3)	2929 (71.4)	
Family history of asthma, n (%)				<0.001
Yes	1195 (27.0)	270 (55.3)	925 (23.9)	
No	2306 (50.5)	129 (27.8)	2177 (53.0)	
Unknown	1214 (22.5)	88 (16.9)	1126 (23.1)	
Physical activity, n (%)				0.645
Not ideal	1265 (24.5)	131 (23.3)	1134 (24.7)	
Ideal	1749 (32.8)	170 (31.2)	1579 (33.0)	
Unknown	1701 (42.7)	186 (45.5)	1515 (42.4)	
Sedentary time, n (%)				0.991
< 3	867 (18.7)	81 (18.4)	786 (18.7)	
3-6	1078 (20.4)	122 (20.2)	956 (20.5)	
≥ 6	1950 (39.5)	204 (40.5)	1746 (39.4)	
Unknown	820 (21.4)	80 (21.0)	740 (21.4)	
Pregnant smoking, n (%)				0.217
Yes	487 (10.6)	64 (13.0)	423 (10.4)	
No	3580 (73.2)	360 (72.4)	3220 (73.3)	
Unknown	648 (16.2)	63 (14.6)	585 (16.3)	
Breast feeding, n (%)				0.003
No	343 (5.7)	24 (3.3)	319 (5.9)	
Yes	1091 (21.2)	80 (16.3)	1011 (21.8)	
Unknown	3281 (73.1)	383 (80.4)	2898 (72.3)	
Low birth weight, n (%)				<0.001
No	3399 (70.5)	323 (65.8)	3076 (71.0)	
Yes	572 (11.1)	93 (16.8)	479 (10.5)	
Unknown	744 (18.4)	71 (17.4)	673 (18.5)	
Vitamin A, mcg, Mean (S.E)	591.13 (12.68)	589.42 (29.91)	591.32 (12.34)	0.944
Vitamin C, mg, Mean (S.E)	69.53 (2.22)	77.53 (4.29)	68.65 (2.23)	0.032
Vitamin D, mcg, Mean (S.E)	5.05 (0.14)	4.81 (0.25)	5.08 (0.14)	0.216
Vitamin E, mg, Mean (S.E)	8.25 (0.24)	8.31 (0.32)	8.25 (0.25)	0.840
Vitamin B1, mg, Mean (S.E)	1.52 (0.01)	1.58 (0.05)	1.52 (0.01)	0.177
Vitamin B2, mg, Mean (S.E)	1.85 (0.02)	1.88 (0.06)	1.85 (0.02)	0.615
Niacin, mg, Mean (S.E)	21.34 (0.27)	23.01 (1.06)	21.15 (0.25)	0.081
Vitamin B6, mg, Mean (S.E))	1.70 (0.03)	1.77 (0.08)	1.69 (0.03)	0.314
Vitamin B12, mcg, Mean (S.E)	5.62 (0.12)	5.47 (0.29)	5.64 (0.12)	0.569
Folic acid, mcg, Mean (S.E)	202.71 (3.20)	221.16 (12.42)	200.67 (3.34)	0.125
Choline, mg, Mean (S.E)	248.44 (3.49)	254.66 (11.34)	247.76 (3.58)	0.557
Vitamin K, mcg, Mean (S.E)	67.22 (1.76)	64.78 (3.53)	67.49 (1.81)	0.440
BMI Z-score, Mean (S.E)	0.62 (0.03)	0.90 (0.07)	0.59 (0.03)	<0.001
Energy intake, kcal, Mean (S.E)	1905.24 (15.23)	2037.80 (60.20)	1890.63 (13.96)	0.018

*S.E* Standard error, *PIR* Poverty-to-income ratio, *BMI* Body mass index

Statistics: t test for the comparation of continuous variable and chi-square test for that of classified variables

### Relationship between multivitamins consumption and childhood asthma

We first screened the covariates associated with childhood asthma (Table 2). The results showed that age, race, family history of asthma, pregnant smoking, breast feeding, low birth weight, BMI Z-score, and energy intake were all significantly associated with childhood asthma (all *P*<0.05). Then we included these covariates into adjustment of the multivariate models.

**Table 2:** Screening of covariates associated with childhood asthma

Variables	OR (95% CI)	*P*
Age		
2-6	Ref	
7-11	1.64 (1.18-2.29	0.005
12-17	1.52 (1.05-2.20)	0.030
Gender		
Male	Ref	
Female	0.81 (0.62-1.06)	0.115
Race		
White	Ref	
Black	1.93 (1.44-2.58)	<0.001
Other	1.01 (0.77-1.33)	0.932
PIR		
<1	Ref	
≥1	0.78 (0.58-1.03)	0.078
Unknown	0.84 (0.47-1.50)	0.538
Household smoke exposure		
No	Ref	
Yes	0.72 (0.29-1.79)	0.469
Unknown	0.64 (0.25-1.61)	0.327
Family history of asthma		
No	Ref	
Yes	4.40 (2.98-6.52)	<0.001
Unknown	1.40 (0.97-2.03)	0.072
Physical activity		
Non-ideal	Ref	
Ideal	1.00 (0.69-1.46)	0.993
Unknow	1.14 (0.77-1.68)	0.503
Sedentary time		
<3	Ref	
3-6	1.00 (0.72-1.39)	0.987
≥6	1.04 (0.70-1.55)	0.826
Unknown	1.00 (0.61-1.62)	0.996
Pregnant smoking		
No	Ref	
Yes	1.40 (1.03-1.91)	0.033
Unknown	1.10 (0.83-1.47)	0.495
Breast feeding		
No	Ref	
Yes	1.34 (0.76-2.36)	0.296
Unknown	1.99 (1.16-3.42)	0.014
Low birth weight		
No	Ref	
Yes	1.73 (1.24-2.40)	0.002
Unknown	1.01 (0.79-1.30)	0.929
BMI Z-score	1.28 (1.13-1.45)	<0.001
Energy intake	1.01 (1.01-1.01)	0.008

*OR* Crude odds ratio, *CI* Confidence interval, *Ref* Reference, *PIR* Poverty-to-income ratio, *BMI* Body mass index

For continuous variables including BMI Z-score and energy intake, the explanation on the association between them and outcomes should be that for each 1-unit increase in BMI Z-score/energy intake, the odds of childhood asthma increased 1.28/1.01

The relationship between multivitamins consumption and childhood asthma was further explored (Table 3). After adjusting for covariates, we only found that for each 1-unit increase in VK consumption, the odds of childhood asthma decreased 0.99 [OR=0.99, 95% CI: (0.99-0.99)].

**Table 3:** Association between multivitamins consumption and childhood asthma

Variables	Crude model		Adjusted model^a^	
	OR (95% CI)	*P*	OR (95% CI)	*P*
Vitamin A	1.00 (1.00-1.00)	0.945	1.00 (1.00-1.00)	0.352
Vitamin C	1.01 (1.01-1.01)	0.029	1.00 (1.00-1.00)	0.134
Vitamin D	0.99 (0.97-1.01)	0.243	0.98 (0.96-1.00)	0.070
Vitamin E	1.00 (0.99-1.01)	0.836	0.99 (0.98-1.00)	0.107
Vitamin B1	1.10 (0.96-1.25)	0.153	0.95 (0.78-1.17)	0.634
Vitamin B2	1.02 (0.93-1.13)	0.604	0.90 (0.79-1.02)	0.090
Niacin	1.01 (1.01-1.02)	0.022	1.00 (0.99-1.02)	0.536
Vitamin B6	1.05 (0.96-1.16)	0.279	0.99 (0.88-1.12)	0.932
Vitamin B12	0.99 (0.97-1.02)	0.579	0.98 (0.95-1.00)	0.081
Folic acid	1.00 (1.00-1.00)	0.086	1.00 (1.00-1.00)	0.412
Choline	1.00 (1.00-1.00)	0.533	1.00 (1.00-1.00)	0.440
Vitamin K	1.00 (1.00-1.00)	0.488	0.99 (0.99-0.99)	0.028

*OR* Odds ratio, *CI* Confidence interval

^a^Adjusted for age, race, family history of asthma, pregnant smoking, BMI Z-score, energy intake, breast feeding, and low birth weight

The explanation on the association between multivitamins consumption and childhood asthma should be that for each 1-unit increase in each multivitamin intake, the odds of childhood asthma increased/decreased the OR value

### Overall effect of dietary multivitamins intake on childhood asthma

**Fig 2:**
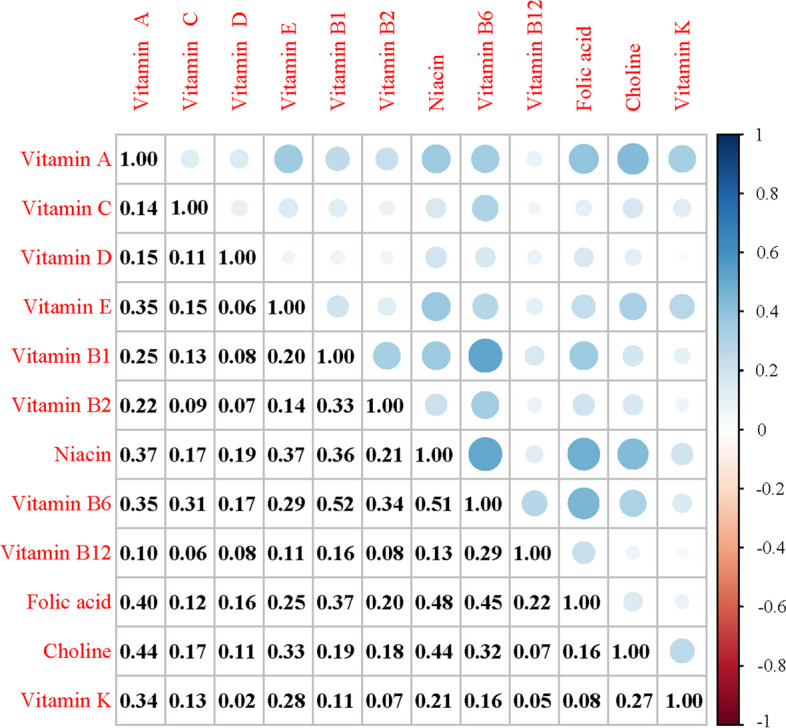
Pearson correlation analysis of different vitamins. The blue color represents the positive association, and the red color represents the negative association. A darker color indicates a stronger association

Figure 2 shows the Pearson correlation analysis of different vitamins. The twelve vitamins were divided into three groups according to the similar values in correlation matrix (Table 4). Specifically, VC, VD, VB12, and VK were grouped together (group PIP=1.00); Choline, VA, VE, and VB2 were grouped together (group PIP=0.88); niacin, VB6, VB1, and folic acid were grouped together (group PIP=0).

**Table 4:** The PIP of each vitamin in and between multivitamin groups

Variables	Group	Group PIP	Cond PIP
Vitamin C	1	1.00	0.8
Vitamin D	1	1.00	0.2
Vitamin B12	1	1.00	0
Vitamin K	1	1.00	0
Choline	2	0.88	0
Vitamin A	2	0.88	0
Vitamin E	2	0.88	0
Vitamin B2	2	0.88	1.0
Niacin	3	0	0
Vitamin B6	3	0	0
Vitamin B1	3	0	0
Folic acid	3	0	0

*PIP* Posterior inclusion probability

**Fig. 3:**
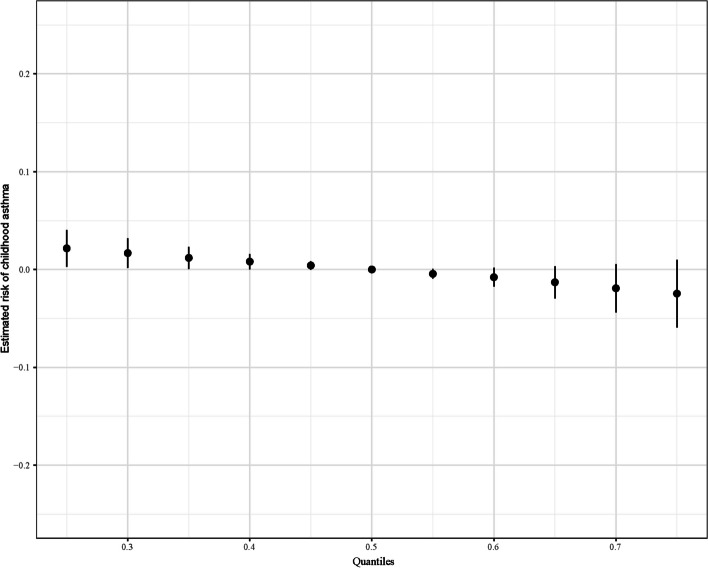
The overall effect of the multivitamins on the estimated risk of childhood asthma by BKMR. The results were assessed by the BKMR model, adjusted for age, race, family history of asthma, pregnant smoking, breast feeding, low birth weight, BMI Z-score, and energy intake

The estimated overall effect of dietary multivitamins on childhood asthma by BKMR was showed in Fig. 3. We first fixed all of the twelve vitamins to their median values, and compared the estimated risk of childhood asthma when these vitamins concentrations at different percentiles (from 0 to 100%). After adjusting for covariates, the multivitamins consumption showed a negative overall effect on the estimated risk of childhood asthma especially when the multivitamin concentrations at their 75th percentile and over.

### Association between single vitamin in the multivitamins and childhood asthma

We also compared the PIP of each single vitamin in and between multivitamin groups in the association with childhood asthma (Table 4). The results showed that in the first group, VC had the most contribution for childhood asthma (Cond PIP=0.8). In both the second group and the third group, VB2 had the most contribution for childhood asthma (Cond PIP=1.0).

**Fig. 4:**
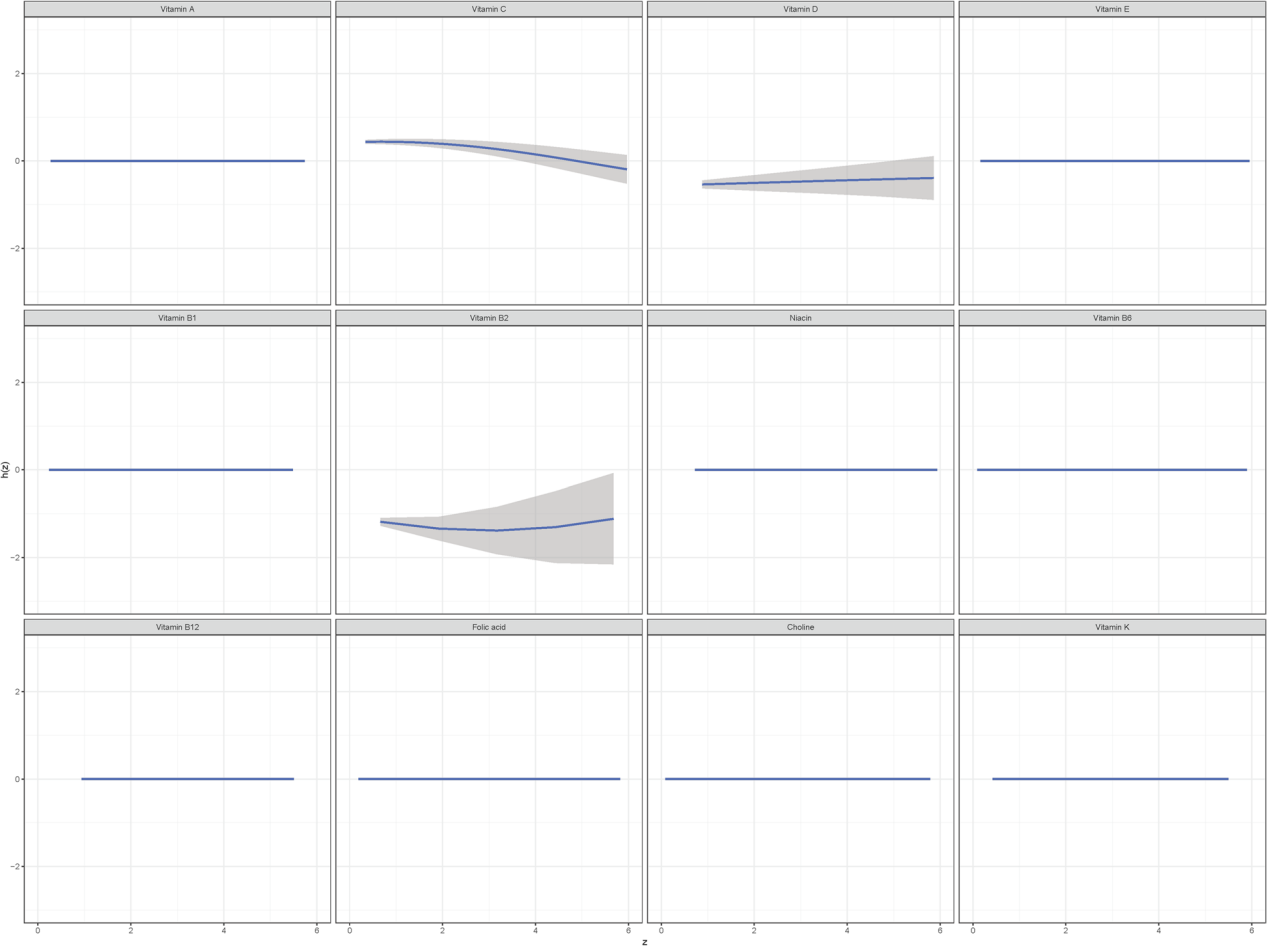
The contribution of each vitamin to the overall effect and its dose-response. h(Z) can be interpreted as the relationship between vitamins and a latent continuous outcome (continuous marker of the binary asthma outcomes). The results were assessed by the BKMR model adjusted for age, race, family history of asthma, pregnant smoking, breast feeding, low birth weight, BMI Z-score, and energy intake

The curves of contribution of each vitamin to the overall effect and its dose-response were also plotted (Fig. 4). When all of the vitamins were fixed to their median values, the odds of childhood asthma increased along with elevated VD and VB2, whereas the odds of childhood asthma decreased along with elevated VC intake.

### Potential interactions between vitamins on childhood asthma

**Fig. 5:**
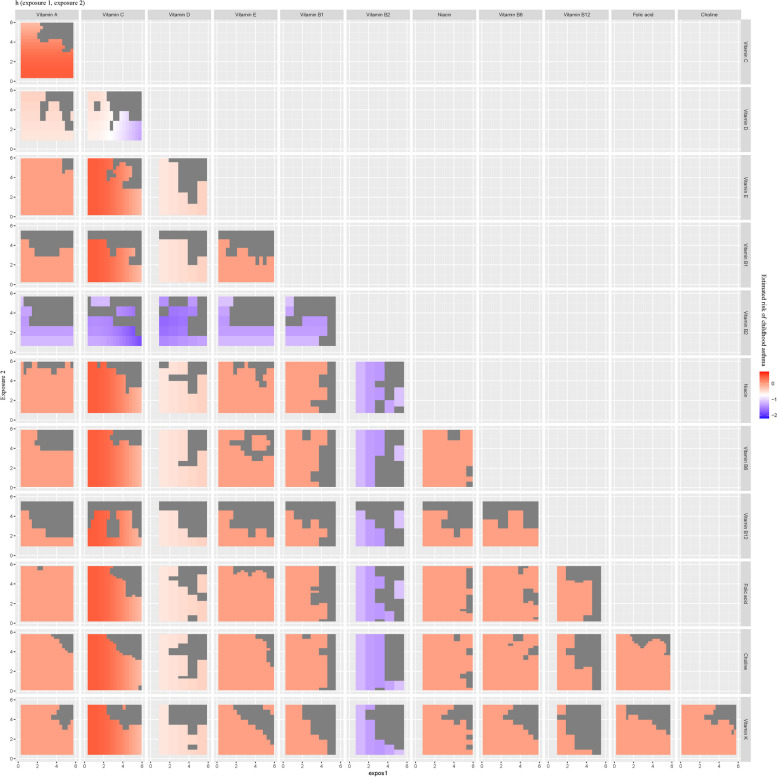
The bivariate exposure–response functions of each two vitamins. Bivariate exposure–response functions for each tow vitamins when other vitamins were fixed at varying (10th, 50th, 90th) percentiles

**Fig. 6:**
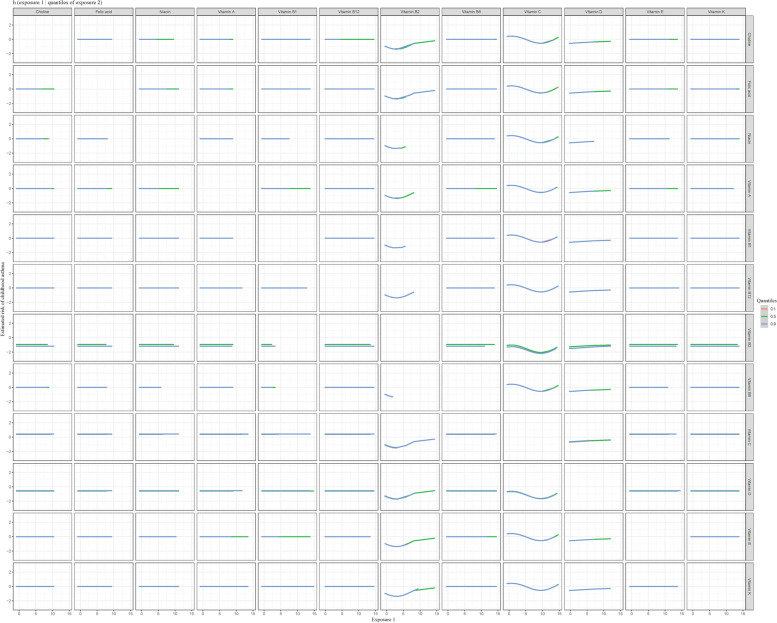
The potential interactions between each two vitamins. Bivariate exposure–response functions for each two vitamins when one of the two vitamins was fixed at varying (10th, 50th, 90th) percentiles and the other vitamins were fixed at the median

To investigate whether there is interaction between every two vitamins among the multivitamins, we plotted the bivariate exposure-response functions curves (Figs. 5 and 6). When fixed one of each two vitamins at the 10th, 50th, and 90th percentiles respectively (with the rest ten vitamins at their median), no potential interaction between every two vitamins of multivitamins in their associations with childhood asthma has been found.

## Discussion

This study explored the association between multivitamins and childhood asthma. We found that elevated dietary multivitamins consumption was overall associated with decreased odds of childhood asthma. To be specific, for each 1-unit increase in VK consumption, the odds of childhood asthma decreased. Among the multivitamins, VD and VB2 had positive associations with childhood asthma, whereas that VC had a negative one. Moreover, no potential interaction between every two vitamins of the multivitamins on the childhood asthma was found.

To the best of our knowledge, no study has explored the relationship between multivitamins intake and asthma in children and adolescents. A Swedish birth cohort study on using of multivitamin supplements in relation to allergic disease in 8 years old children found no relationship between current use of multivitamins and the risk of allergic disease [25]. However, Milner et al. [26] showed that early vitamin supplementation was linked to an increased risk for asthma in Black children. Another study investigated the association between multivitamin supplements intake and asthma development in Norwegian adults, which also demonstrated that regular multivitamin supplements was related to an increased OR for incident asthma [27]. Similarly, in the current study, we found an overall negative effect of dietary multivitamins intake on the odds of asthma in children and adolescents. Childhood asthma is an allergenic disease that influenced by both genetic and environmental factors [28]. Comparing with previous studies, participants in our research had different age, race, and multivitamins intake pattern, which may all affect the influencing factors related to childhood asthma. The average age of Children with asthma in our study was 10 years old, and 1,065 (13.0%) of them were Black. Aging directly and significantly leads to future exacerbations in asthma [29]. A study has reported that Black and Latinx Americans had a higher prevalence and greater morbidity of asthma than the White Americans [30]. Racial/racial inequities such as socioeconomic status, environmental exposures, and health care access/quality are social determinants of health, which result in excess burden of asthma prevalence/incidence [30, 31]. Herein, we have adjusted these covariates and also found an approximately negative relationship between multivitamins consumption and childhood asthma, which relatively made our results more robust.

Among the multivitamins, we compared the PIP of the effect of each vitamin on asthma, and the results showed that VC, VD, and VB2 were the mainly contributors. Also, when all the vitamins were fixed to the median, dietary VD and VB2 intakes were positively associated with the odds of childhood asthma, while that VK had a negatively relationship. The role of VC as an antioxidant vitamin in allergic diseases such as asthma has been extensively studied [32, 33]. A systematic review and meta-analysis of randomized controlled trials by Kumar et al. [34] suggested that VD supplementation might not have any protective effect in childhood asthma. Gale et al. [35] found that exposure to maternal concentrations of 25(OH)-VD >75 nmol/L compared to those <30 nmol/L in late pregnancy had an increased risk of offspring asthma at 9 years old. However, some other studies showed opposite results that higher VD concentration may reduce the risk of developing childhood asthma [36, 37]. In the pathogenesis of childhood asthma, Lung helper T cell 2 (Th2) causes airway hyper-responsiveness and remodeling through inflammatory pathways [38]. Supplement with VD and VC increases in the production of pro-inflammatory cytokines, and can reduce respiratory infections, which suggested they can enhance the immune response, thereby reducing the risk of asthma [10, 39]. However, further studies are needed to make these causal relationships clearer. VB2, namely riboflavin, is the vitamin that most frequently induces allergic reactions [40]. VB2 directly affects both vasodilatation and bronchoconstriction in conjunction with the triggering of mast cell degranulation [41]. High level of VB2 may result in the bronchial hyperresponsiveness and contribute to the development of childhood asthma. On the contrary, we found an opposite relationship between VK intake and the odds of asthma. In fact, menaquinone (vitamin K2) was used as an effective bronchial asthma drug [42]. Shibata et al. [43] found that the VK-dependent soluble protein known as growth arrest-specific protein (Gas) 6 levels exerted in allergic airway disease. According to our results, although it is statistically significant, the estimated association of asthma with VK consumption was very small (OR=0.99), indicating that children and adolescents with high-risk of asthma need to focus on the regular examination on VK concentration, and timely supplement appropriate dietary VK under medical supervision in clinical practice, which may further be beneficial to prevention of childhood asthma. However, the opportune prevention dosage as well as the specific mechanism of the potential protective effect of VK on childhood asthma is still needed further exploration.

Through the BKMR model, we also investigated the interactions between every two vitamins among the multivitamins on childhood asthma. When fixed one of the two vitamins at the 10th, 50th, and 90th percentiles respectively (with the rest ten vitamins at the median), no potential interactions between these two vitamins on childhood asthma was found. Several studies have reported potential interactions of different vitamins and the underlying mechanisms in different physiological process. The B-group vitamins including B2, B6, folic acid, betaine, choline, and B12 are reported play roles in human DNA methylation [44]. Folic acid, VB12, and choline are a source of methyl donors for DNA methylation, and have been extensively studied in asthma [45]. Besides, the BKMR is a recent developed approach for estimating the health effects of multi-pollutant mixtures, and is of increasing interest in environmental epidemiology [46]. In addition to multi-pollutant mixtures, BKMR has also been used in studying the exposure to some conventional trace elements. Predictive process method of BKMR leads to a substantial reduction in the runtime, without a major decrease in accuracy, and in the setting of a larger number of exposures and a dichotomous outcome, so that the probit BKMR implementation is able to correctly identify the variables included in the exposure-response function [46].

There are strengths and limitations in this study. Our study sample size was large and representative due to the data was extracted from the NHANES database. We used the BKMR model to explore the overall effect of multivitamins consumption on childhood asthma, which may provide some references for the assessment and guidance of dietary multivitamins supplement in children and adolescents with high-risk of asthma. Some limitations of this study must be considered when interpreting the results. This is a cross-sectional study that could not clarify the causal relationship between multivitamins intake and childhood asthma. Data of vitamins intake were collected by 24-hour dietary recall, which may cause recalling bias. Besides, the 24-hour dietary recall only reflected recent dietary trends, but the long-term dietary status was unavailable. The diagnosis of asthma was self-reported, which perhaps may underestimate the actual prevalence of childhood asthma. Moreover, due to the limitation of database, the impact of under-reporting of those with missing data cannot be ignored.

## Conclusion

Dietary consumption of multivitamins was overall negatively associated with the odds of childhood asthma. Our findings recommended that children and adolescents should increase the intake of VC-rich foods, whereas control the dietary consumption of VD and VB2 in daily life.

## Data Availability

The datasets used and/or analyzed during the current study are available from the NHANES database, https://www.cdc.gov/nchs/nhanes/index.htm.

## Abbreviations

VC: Vitamin C
VE: Vitamin E
ROS: Reactive oxygen species
VB12: Vitamin B12
NHANES: National Health and Nutrition Examination Survey
NCHS: National Center for Health Statistics
MEC: Mobile exam centers
BMI: Body mass index
IRB: Institutional Review Board
CDC: Centers for Disease Control and Prevention
VA: Vitamin A
VD: Vitamin D
VB1: Vitamin B1
VB2: Vitamin B2
VB6: Vitamin B6
VK: Vitamin K
MET: Metabolic equivalent
PAQ: Physical activity questionnaire
Mean±SD: Means and standard deviation
MEC: Mobile examination center
PSUs: Pseudo-primary sampling units
